# Transcriptome analysis of the almond moth, *Cadra cautella*, female abdominal tissues and identification of reproduction control genes

**DOI:** 10.1186/s12864-019-6130-2

**Published:** 2019-11-21

**Authors:** Mureed Husain, Muhammad Tufail, Khalid Mehmood, Khawaja Ghulam Rasool, Abdulrahman Saad Aldawood

**Affiliations:** 1 Economic Entomology Research Unit, Department of Plant Protection, College of Food and Agriculture Sciences, King Saud University, 2460, Riyadh, 11451 Kingdom of Saudi Arabia; 2 Ghazi University, Dera Ghazi Khan, Punjab Pakistan

**Keywords:** *Cadra cautella*, Next-generation sequencing, Female abdominal tissues, Transcriptome, Reproduction

## Abstract

**Background:**

The almond moth, *Cadra cautella* is a destructive pest of stored food commodities including dates that causes severe economic losses for the farming community worldwide. To date, no genetic information related to the molecular mechanism/strategies of its reproduction is available. Thus, transcriptome analysis of *C*. *cautella* female abdominal tissues was performed via next-generation sequencing (NGS) to recognize the genes responsible for reproduction.

**Results:**

The NGS was performed with an Illumina Hiseq 2000 sequencer (Beijing Genomics Institute: BGI). From the transcriptome data, 9,804,804,120 nucleotides were generated and their assemblage resulted in 62,687 unigenes. The functional annotation analyses done by different databases, annotated, 27,836 unigenes in total. The transcriptome data of *C. cautella* female abdominal tissue was submitted to the National Center for Biotechnology Information (accession no: PRJNA484692). The transcriptome analysis yielded several genes responsible for *C. cautella* reproduction including six *Vg* gene transcripts. Among the six *Vg* gene transcripts, only one was highly expressed with 3234.95 FPKM value (fragments per kilobase per million mapped reads) that was much higher than that of the other five transcripts. Higher differences in the expression level of the six *Vg* transcripts were confirmed by running the RT-PCR using gene specific primers, where the expression was observed only in one transcript it was named as the *CcVg*.

**Conclusions:**

This is the first study to explore *C. cautella* reproduction control genes and it might be supportive to explore the reproduction mechanism in this pest at the molecular level. The NGS based transcriptome pool is valuable to study the functional genomics and will support to design biotech-based management strategies for *C. cautella*.

## Background

Date palm, *Phoenix dactylifera* is an important fruit tree of the Arabian Peninsula and temperate regions worldwide [1]. In hot dry regions globally, dates have a very important history and are considered one of the most important nutritional fruits. Dates can be consumed in many ways, such as eaten directly as fresh dates, eaten as dried dates, and also used in the preparation of date cookies, date paste, date syrup, and many other products. Additionally, dates have a very important medicinal value as they contain a rich source of minerals [2]. The presence of amino acids, flavonoids, steroids, anti-oxidants, anti-inflammatory, and anticancer elements in the flesh highlights the medicinal and nutritional importance of dates [3, 4]. The by-products of dates are used for the production of organic acids, antibiotics, and fermented yeast. In the Gulf region, the populace prefer to consumes a certain quantity of dates [5].

Several devastating pests can infest date fruits causing great economic losses. These pests include the almond moth, *Cadra cautella* (Walker) (Lepidoptera: Pyralidae) and the sawtoothed grain beetle, *Oryzaephilus surinamensis* [1]. In the Middle East as well as in many other regions of the world, *C. cautella* is a destructive polyphagous storage pest of date fruits, cereals, dried fruits, ground nuts, and maize [6–8]. The life cycle of *C. cautella* is short with many generations per year and a single female can produce 213 and 422 eggs/female, when reared on artificial diet and “khodari” date fruits, respectively [7, 9–12].

The moth, *C. cautella* infests date fruits both in the field as well as in the warehouses and deteriorates the quantity and quality of dates, which leads to trade restrictions. Many countries enforce strict quarantine limitations, which bound the world trade in agricultural produce [13]. The control of *C. cautella* mostly depends on fumigation with methyl bromide and phosphine gas, which are effective and inexpensive and have been widely applied over the last few decades. However, recently the use of such control treatments have been questioned because the excessive use of these chemicals poses environmental concerns for human health as well as the phosphine resistance that has been reported in several stored product insect species [14–16]. In addition, methyl bromide, that was an efficient and cost effective fumigant; has been declared an ozone depleting chemical and has been phased out of production and use [17].

Several studies have reported on the basic ecological and biological characteristics of *C. cautella* [11, 18–20]. Therefore, there is an urgent need to develop environmentally friendly strategies to manage this serious pest. However, the molecular mechanism of its reproduction remains unknown. Over the last two decades, genomes of different insects have been sequenced. Genes related to reproduction, physiology, and sex pheromone biosynthesis and their receptors have been intensively studied for further analysis [21–26]. Thus, the objective of the present study was to identify the reproduction control genes through transcriptome data analysis especially the vitellogenin (Vg). Vg is the key component of egg yolk protein, synthesized extra-ovarially in the fat body tissues, and transported to the developing oocytes where it is internalized in the egg by the VgR and serves as a nutrient source for the developing embryo. Vg and VgR have been reported at the genetic and molecular level in many insect species [21, 22, 27–31].

The transcriptome is an entire set of transcripts in a cell, tissue, or organism. De novo transcriptome sequencing is a method of creating a transcriptome profile via the Illumina HiSeq 2000/2500 platform [32]. Next-generation sequencing (NGS), can extensively explore the structure and provide indication about functional role of a particular gene product in a given tissue without the aid of any reference genome [33, 34]. The NGS is an analytical technique that sequences RNA molecules with a large number of reads [35–37]. Transcriptome analysis has been used to study fatal diseases in humans, plants, and other organisms [38–40]. Transcriptomes from many insect species have been sequenced such as the silkworm, *Bombyx mori*, red flour beetle, *Tribolium castaneum*, and oriental fruit fly, *Bactrocera dorsalis* [41–43].

Sequencing of *C. cautella* abdominal tissues transcriptome would clarify the reproduction strategies of at the molecular level.

To the best of our knowledge, the present study is the first to report on the transcriptome analysis of *C. cautella* abdominal tissues, provides evidence-based knowledge to facilitate the development of future eco-friendly management strategies for this pest.

## Results

### *Cadra cautella* transcriptome sequencing and sequence assembly

A library of *C. cautella* adult female abdominal tissue was sequenced by the Illumina Hiseq 2000 system. The transcriptome generated raw reads, these reads were cleaned with the help of filter-fq software (version: internal filter_fq software of BGI). The de novo assembly detected 62,687 unigenes. The details of unigenes total length, average length, and N50 is presented in (Additional file 1: Table S1).

### Structural and functional annotation of unigenes

For functional annotation analysis, we obtained 25,880, 15,432, 17,738, 16,106, 8828, 9494 unigenes, which annotated to the NR, NT, Swiss-Prot, KEGG, COG, and GO databases, respectively. The total annotated unigenes were 27,836 (Table 1). For protein coding region prediction analysis, the number of coding DNA sequence (CDS) that mapped to the protein database was 25,715, whereas the number of predicted CDS was 2719 (Additional file 3: Table S2).

**Table 1:** Summary of annotated unigenes obtained from *Cadra cautella* female abdominal tissue transcriptome analysis

Annotated databases	Number of unigenes	Percentage (%)
NR	25,880	41.28
NT	15,432	24.61
Swiss-Prot	17,738	28.29
KEGG	16,106	25.69
COG	8, 828	14.08
GO	9, 494	15.14
Total	27, 836	

**Fig. 1:**
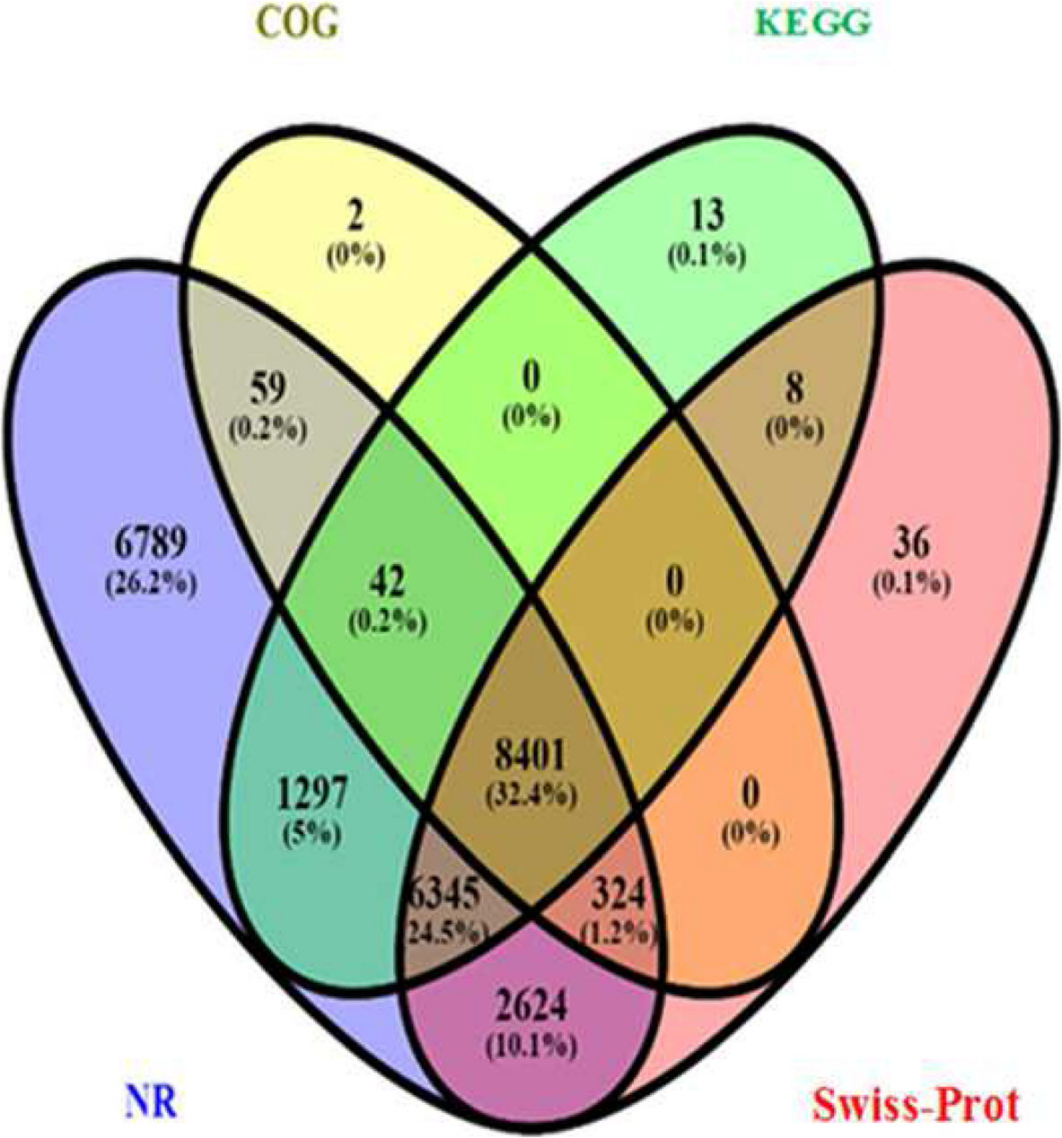
Schematic presentation of Cadra cautella female abdominal tissue transcripts annotated in different protein databases (e-value < 0.00001)

Among the unigenes, 6789, 2, 13, and 36 were annotated exclusively to the NR, COG, KEGG, and Swiss-Prot protein databases, respectively, with 1297 unigenes annotated using both the NR and KEGG databases. In addition, 42 unigenes were commonly annotated using the NR, COG, and KEGG databases whereas no unigenes were commonly annotated using the KEGG and COG protein databases. Furthermore, 8401 common elements were annotated in the NR, COG, KEGG, and Swiss-Prot databases (Fig. 1).

**Fig. 2:**
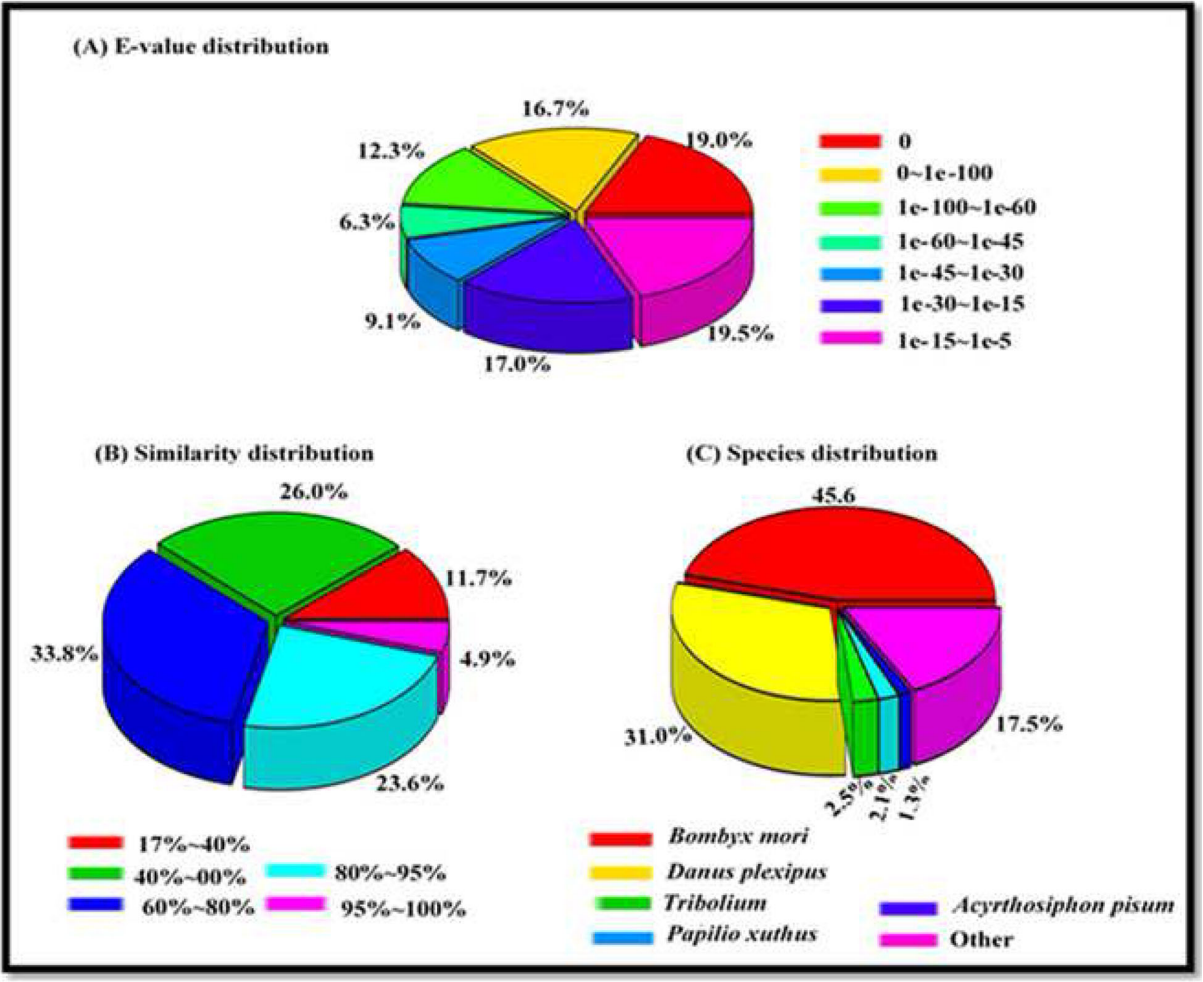
Proportional distribution of e-value, sequence similarity, and species distribution unigenes against the non-redundant protein (NR) database

A total of 27,836 unigenes sequences shared some similarity to known genes from the National Center for Biotechnology Information (NCBI) database. The ranges in e-value and sequence similarity of the top hits in the NR database were comparable, with 49% (e-value of 0 to 60) and 28.5% (100–80%), respectively, of the sequences possessing homology (Fig. 2a, b). On a species basis, the highest proportion of matching sequences in the NR database were derived from *Bombyx mori* (45.59%), followed by *Danaus plexippus* (31%) (Fig. 2c).

**Fig. 3:**
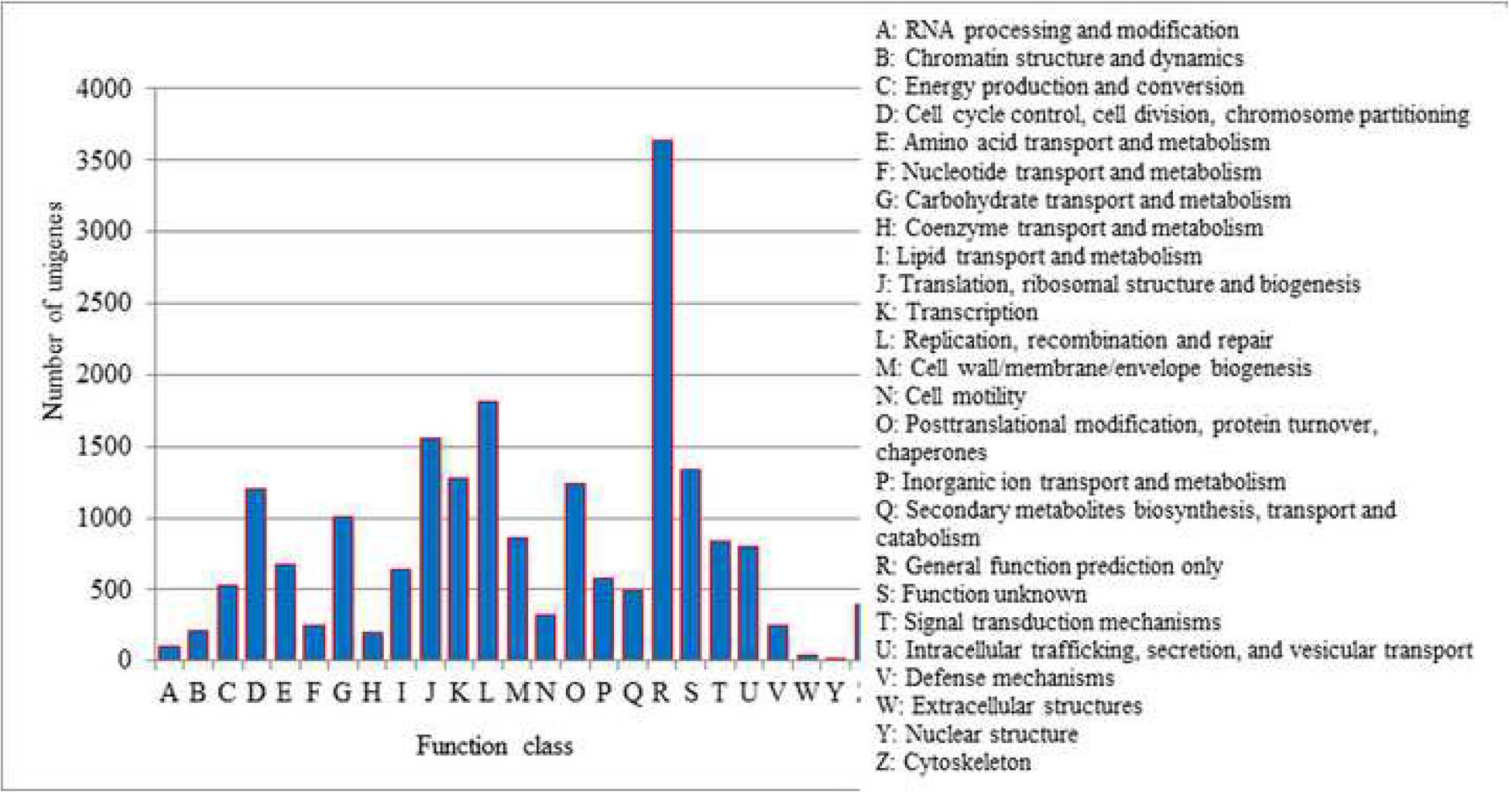
COG functional classification of unigenes from Cadra cautella female abdominal tissue transcriptome. The horizontal coordinates represent the functional classes identified using COG analysis and the vertical coordinates shows the numbers of unigenes in each class. The functions of each class are provide in the notation on the right

Functional annotation was assigned using the protein (NR and Swiss-Prot), COG, and GO databases. BLASTX was employed to identify related sequences in the protein databases. The COG database attempts to classify proteins from completely sequenced genomes on the basis of the orthology concept. The COG analysis permitted the functional classification of 8828 of the unigenes. Among these genes, the peak regularly recognized classes including “general function” (3636, 41.18%), followed by “replication, recombination, and repair” (1816, 20.57%), “translation, ribosomal structure, and biogenesis” (1562, 17.69%), “function unknown” (1342, 15.20%), “transcription” (1278, 14.47%), and “posttranslational modification, protein turnover, and chaperones” (1237, 14.01%) (Fig. 3).

**Fig. 4:**
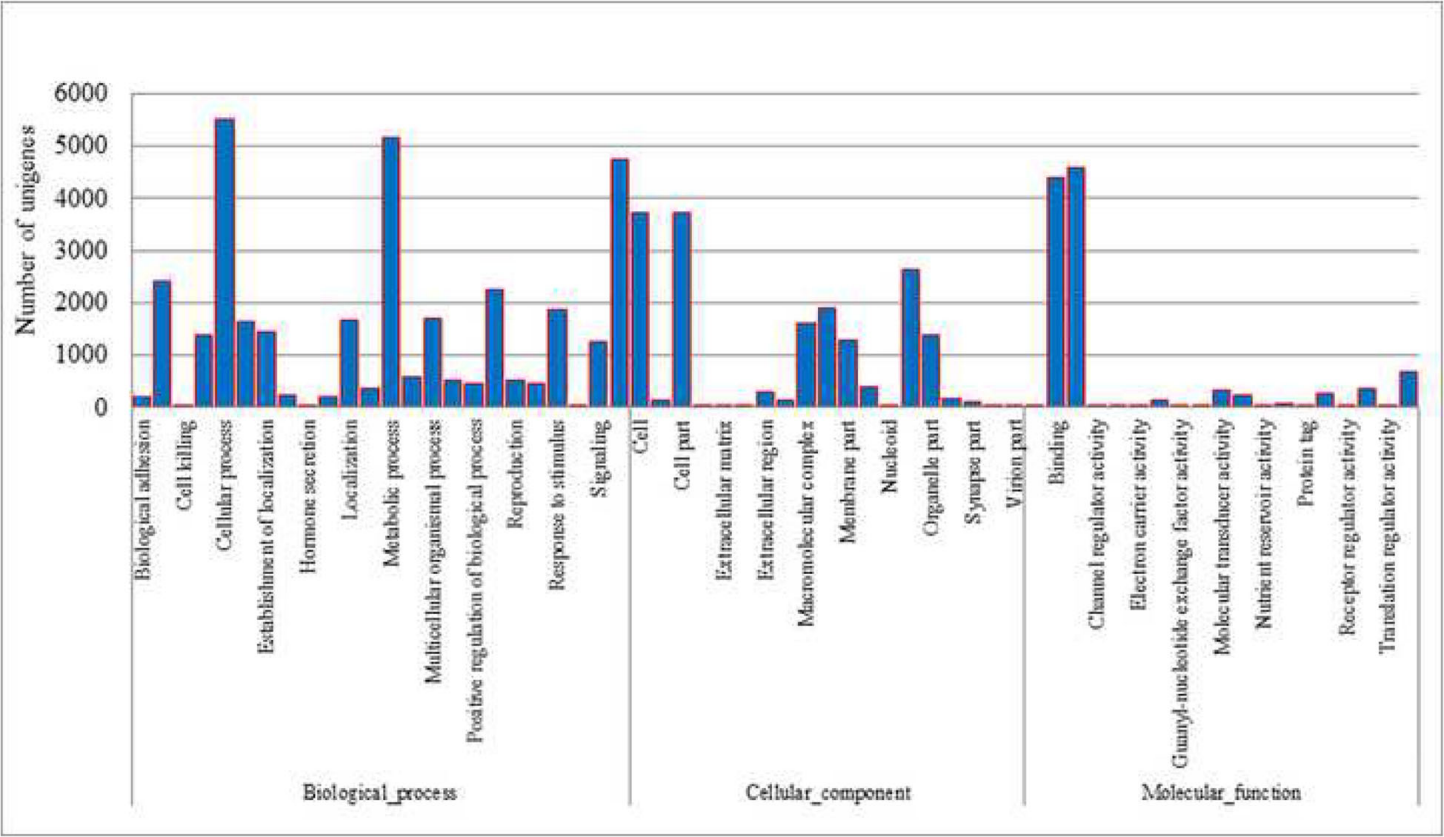
GO functional classification of unigenes identified from Cadra cautella female abdominal tissue transcriptome. The horizontal coordinates represent the functional classes identified using GO analysis and the vertical coordinates show the numbers of unigenes in each class

Functionally categorized genes of *C. cautella* were assigned GO terms for each assembled unigenes [44]. The unigenes were placed in three main GO categories: biological process (34,770, 55.46%), cellular component (17,661, 28.17%), and molecular function (11,232, 17.91%). These GO terms were additionally sectioned into 62 sub-categories. NR annotation was given the type of “biological process” and, within this ontology, the three most common functions were “biogenesis” (5521, 15.27%), “metabolic process” (5177, 14.88%), and “single-organism process” (4731, 13.60%). At the level of cellular components, the three most common functions were “cell part” to 3714 unigenes (21.02%), “cell” to 3714 unigenes (21.02%), and “organelle” to 2637 unigenes (14.93). Whereas within the ontology of molecular functions, “catalytic activity” (4574, 40.72%) and “binding” (4380, 38.99%) proteins made up the majority of the unigenes (Fig. 4).

### Protein coding region prediction

**Fig. 5:**
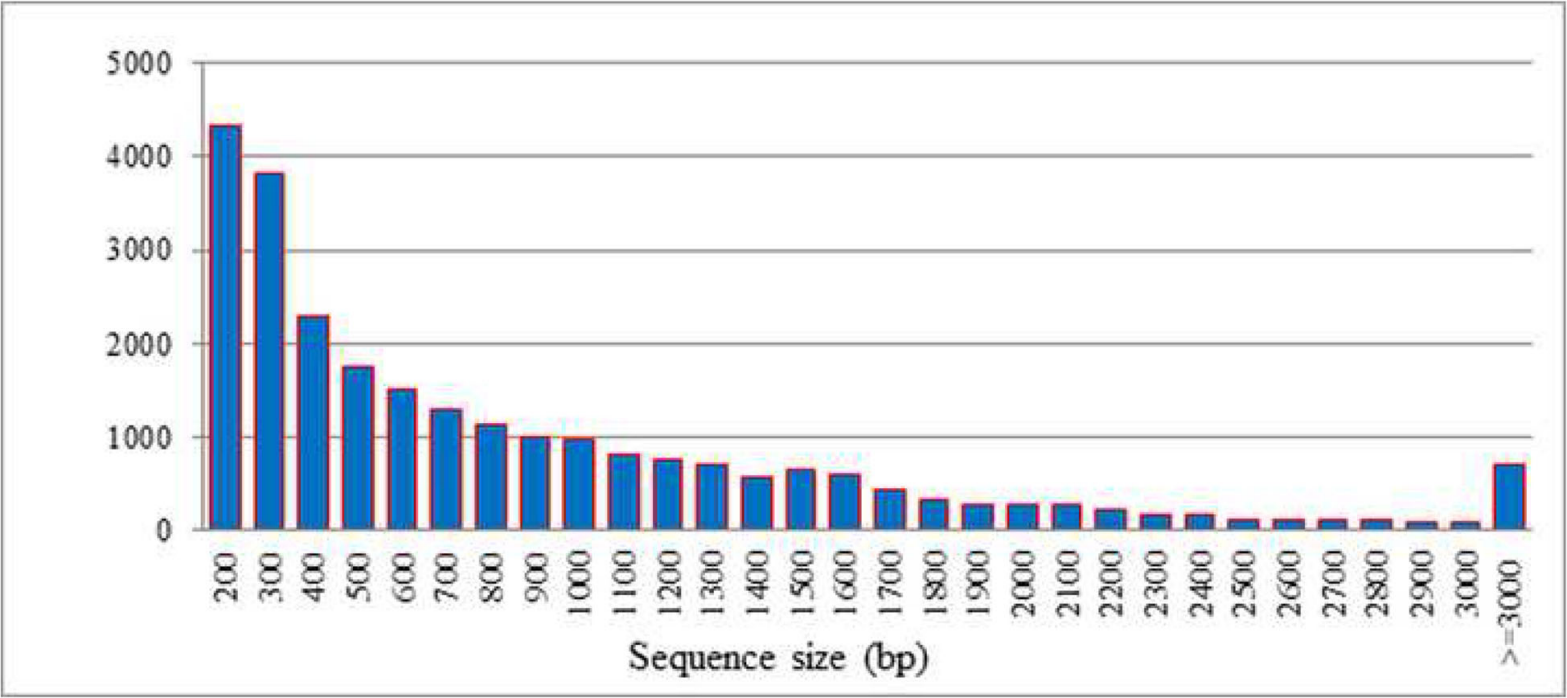
Length distribution of protein-coding sequence from Cadra cautella female abdominal tissue transcriptome. The horizontal axis shows the length and the vertical axis shows the numbers of unigenes with a given length

**Fig. 6:**
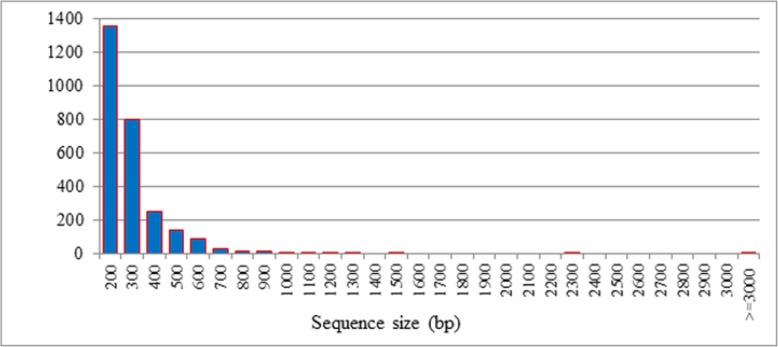
Length distribution of ESTScan sequences from Cadra cautella female abdominal tissue transcriptome. The horizontal axis shows the length while the vertical axis shows the numbers of unigenes with a given length

Unigenes were aligned by BLASTX (e-value < 0.00001) to protein databases in the following order: NR, Swiss-Prot, KEGG, and COG. Proteins with the highest ranks in the BLAST results were taken to decide the coding region sequences of unigenes, and the coding region sequences were translated into amino sequences. Unigenes that could not be aligned to any database were scanned by ESTScan (Version = V3.0.2) to predict the protein coding region, which is very important to determine the sequence direction (5′ – > 3′). The number of CDS that mapped to the protein databases was 25,715, whereas the ESTScan predicted that the CDS would be 2719 unigenes. The total number of CDS obtained in the study was 28,434 (Additional file 3: Table S2). The prediction of the protein coding region is very important to determine the accurate functioning of a gene, because the DNA is a long molecule that carries genes and these genes contain introns and exons. The exons are the only segments of a gene that carries the code for protein formation. The protein-coding sequenc and distribution of ESTScan sequences from *Cadra cautella* female abdominal tissue transcriptome are presented in (Figs. 5 and 6).

### Most highly abundant transcripts in the *Cadra cautella* female abdominal tissue

The transcripts that were most highly expressed in the *C. cautella* adult female abdominal tissues are presented in Table 2. The highly abundant transcripts were yolk polypeptide 2 and follicular epithelium yolk protein subunits with FPKM values of 19,538.56 and 6939.47, respectively. Moreover, apolipophorin III and *Vg* genes were also among the highly expressed transcripts in the *C. cautella* female abdominal tissue with 4262.26 and 3234.95 FPKM values, respectively. The abundance of the reproduction control genes and yolk polypeptide encoding transcripts in the data reflects their key role in the development of future embryos inside the eggs.

**Table 2:** Most highly abundant transcripts detected by transcriptome analysis in the *Cadra cautella* adult female abdominal tissue

Gene ID	Accession no.	Sequence description	Species	Accession no of the reference species	NR score	E-value	FPKM
Unigene20799	MF067302	Yolk polypeptide 2	*Plodia interpunctella*	AF063014.1	1089	0	19,538. 5683
Unigene19939	MF067301	Follicular epithelium yolk protein subunit	*Plodia interpunctella*	AF092741.1	490.3	1.00E-136	6939.4765
CL7565.Contig1	MF067300	40S ribosomal protein S23	*Papilio dardanus*	AJ783764.1	294.7	4.00E-78	5124.8472
Unigene16013	MF067299	Apolipophorin III	*Trichoplusia ni*	EU016400.1	265	4.00E-69	4262.2626
CL3689.Contig2	MF067298	Hypothetical protein OXYTRI_13058	*Oxytricha trifallax*	AMCR01020474.1	70.9	1.00E-09	3802.8071
CL9580.Contig2	MF067297	Heat shock 70 kda cognate protein	*Ostrinia furnacalis*	HQ434763.2	1274.6	0	3756.1282
CL1864.Contig2	ALN38805	Vitellogenin	*Corcyra cephalonica*	KJ540279.1	2169.8	0	3234.9556
Unigene18608	MF067296	Alpha-crystallin cognate protein 25	*Plodia interpunctella*	U94328.1	325.5	3.00E-87	3193.8302
CL965.Contig1	MF067295	90-kda heat shock protein HSP83	*Spodoptera frugiperda*	AF254880.1	1393.6	0	3019.4783
CL3705.Contig1	MF067294	Ribosomal protein L10	*Heliconius melpomene cythera*	JF265063.1	451.8	3.00E-125	2841.0602
Unigene16022	MF067293	Ribosomal protein S11	*Heliothis virescens*	AF379640.1	307.4	3.00E-82	2756.4932
Unigene26979	MF067292	Ribosomal protein L8	*Manduca sexta*	GU084298.1	524.6	5.00E-147	2625.6948
Unigene15928	MF067291	Ribosomal protein S8	*Heliconius melpomene cythera*	JF265021.1	408.3	3.00E-112	2610.3604
Unigene12496	MF067290	Cytochrome c oxidase subunit III	*Ephestia kuehniella*	KU877167.1	432.9	4.00E-119	2605.1013
Unigene16016	MF067289	Ribosomal protein S2	*Bombyx mori*	AAV34857.1	531.9	3.00E-149	2600.3979
Unigene14500	MF067288	Ribosomal protein	*Danaus plexippus*	EHJ67142.1	308.9	1.00E-82	2596.9248

### Identification of reproduction control genes from *Cadra cautella* female abdominal tissue

By means of BLASTX, almost 57 genes potentially responsible for *C. cautella* reproduction were identified from the transcriptome analysis of female abdominal tissue. The genes identified were *Vg*, *VgR*, and lipid carrier protein (apolipophorin), sulfur containing amino acids carrying proteins that enhance vitellogenesis (hexamerins) and egg shell protein (chorion). All of these genes were submitted to NCBI and their accession numbers obtained (see Table 3). The details regarding FPKM values, blast hit score, putative identification of the gene, and resemblance with closely related species are presented in Table 3. There were also the transcripts that encode very important proteins and enzymes that play a role in development. The identification of the juvenile hormone and ecdysone receptor might be a very important addition to study the reproductive development in this pest, because these two genes are responsible for regulating many aspects of arthropods life cycles. Insect development and reproduction are mainly linked to the fluctuating levels of juvenile hormone and ecdysone.

**Table 3:** Putative reproduction control genes obtained from transcriptome analysis of *Cadra cautella* adult female abdominal tissue

Unigene	Accession no.	Putative identification	Reference species	Accession no of the reference species	Blast Hit score	E-value	FPKM
CL1864.Contig2	ALN38805	Vitellogenin	*Corcyra cephalonica*	KJ540279.1	2169.8	0	3234.95
Unigene24723	KY924790	Chorion class B protein	*Lymantria dispar*	AAA67868.1	72.8	1.00E-11	1639.6571
CL3668.Contig1	KY924784	Minus strand apolipophorin	*Galleria mellonella*	AAT76806.1	1520	0	341.35
Unigene8987	KY924789	Hexamerin storage pinsp1	*Plodia interpunctella*	AAK71136.1	1331.2	0	286.675
CL5610.Contig1	KY963162	Vitellogenin receptor	*Helicoverpa armigera*	AGF33811.2	2102.4	0	96.4638
CL5405.Contig1	KY938808	Juvenile hormone binding protein	*Galleria mellonella*	AAN06604.3	238	1.00E-60	80.7699
Unigene26649	KY938817	Phosphatidylinositol-binding clathrin assembly protein LAP-like	*Bombyx mori*	XP_004923321.1	639.8	0	47.2175
CL6527.Contig1	KY938823	Minus strand apolipoprotein of lipid transfer particle-I/II	*Bombyx mori*	BAN58736.1	2842.4	0	45.3994
CL1973.Contig1	KY938814	Ecdysone oxidase	*Bombyx mori*	NP_001243996.1	697.2	0	41.0707
CL1019.Contig1	KY938819	Minus strand dynein heavy chain, cytoplasmic-like	*Bombyx mori*	XP_004929769.1	5079.2	0	40.4356
Unigene18933	KY938834	Alpha-2-macroglobulin receptor-associated protein-like isoform X1	*Bombyx mori*	XP_004923555.1	519.6	3.00E-145	40.0091
CL5671.Contig1	KY938809	Juvenile hormone esterase binding protein	*Manduca sexta*	AAD38067.1	420.2	1.00E-115	36.2405
Unigene20565	KY938839	Hydroxymethylglutaryl-CoA lyase	*Danaus plexippus*	EHJ74310.1	541.6	7.00E-152	33.912
CL7480.Contig1	KY924787	Lipophorin receptor	*Galleria mellonella*	ABF20542.1	1858.2	0	23.9231
CL2713.Contig2	KY924788	Hexamerin 2	*Corcyra cephalonica*	AAG44960.1	887.9	0	23.4974
CL3993.Contig1	KY938806	Juvenile hormone epoxide hydrolase	*Bombyx mori*	Q25489.1	686.4	0	23.2035
Unigene26355	KY938818	Clathrin heavy chain	*Danaus plexippus*	EHJ79063.1	2886.7	0	22.6358
Unigene3611	KY924785	Apolipophorin precursor protein	*Bombyx mori*	BAK82317.1	1688.3	0	22.6224
CL6755.Contig1	KY938837	3-hydroxy-3-methylglutaryl-CoA synthase	*Bombyx mori*	NP_001093297.1	785.8	0	19.3691
Unigene16484	KY938810	Juvenile hormone diol kinase	*Danaus plexippus*	EHJ71313.1	293.9	8.00E-78	18.9053
Unigene24962	KY963165	Rab5 GDP/GTP exchange factor-like	*Bombyx mori*	XP_004921576.1	632.1	4.00E-179	18.4215
Unigene23352	KY963168	Putative gonadotropin inducible transcription factor	*Danaus plexippus*	EHJ67957.1	662.5	0	13.0645
Unigene23138	KY938826	Putative myosin light chain kinase	*Danaus plexippus*	EHJ74039.1	305.1	2.00E-80	11.2394
Unigene20850	KY963164	Minus strand dopamine receptor-like	*Bombyx mori*	XP_004928935.1	728.4	0	9.2798
Unigene3617	KY938816	Ecdysone-induced protein 78C	*Danaus plexippus*	EHJ66672.1|	430.6	3.00E-118	8.7132
Unigene19888	KY827830	Vitellogenin	*Danaus plexippus*	EHJ74327.1	578.9	4.00E-163	6.3437
Unigene18811	KY938835	Putative lysozyme	*Bombyx mori*	ADA67927.1	311.6	1.00E-82	6.1371
CL9317.Contig1	KY924783	Apolipophorins	*Danaus plexippus*	EHJ68005.1	94.4	1.00E-16	5.0993
CL7042.Contig2	KY938830	Putative leucine-rich transmembrane protein	*Danaus plexippus*	EHJ76329.1	1500.3	0	4.8844
Unigene16226	KY938833	Thyroglobulin-like isoform X1	*Bombyx mori*	XP_004932955.1	485.3	4.00E-135	4.5646
CL4967.Contig1	KY938807	Juvenile hormone esterase 1 precursor	*Bombyx mori*	NP_001037027.1	602.4	1.00E-169	4.0408
Unigene763	KY938815	3-dehydroecdysone 3beta-reductase	*Danaus plexippus*	EHJ66291.1	488.4	5.00E-136	3.9411
Unigene22352	KY938824	Minus strand prothoracicotropic hormone preproprotein	*Bombyx mori*	NP_001037349.1	190.7	2.00E-46	3.8727
Unigene1800	KY938821	Minus strand endoprotease furin	*Spodoptera frugiperda*	CAA93116.1	100.1	9.00E-19	3.6173
CL1191.Contig2	KY924792	Cytosolic juvenile hormone binding protein	*Bombyx mori*	NP_001037668.1	500.7	2.00E-139	3.4274
Unigene22948	KY938827	Putative myosin	*Danaus plexippus*	EHJ78713.1	93.2	7.00E-17	3.1837
Unigene9111	KY827829	Vitellogenin	*Anthonomus grandis*	AAA27740.1	1056.2	0	3.0956
CL3721.Contig2	KY963166	Minus strand Rab7	*Helicoverpa zea*	ADX66426.1	382.1	1.00E-103	2.8178
CL167.Contig2	KY938812	Minus strand ecdysteroid 22-kinase	*Danaus plexippus*	EHJ76354.1	315.8	7.00E-84	2.6044
CL121.Contig1	KY938811	Ecdysone-inducible protein	*Galleria mellonella*	AAA19579.1	1353.2	0	2.4638
Unigene18146	KY924786	Apolipophorins	*Manduca sexta*	Q25490.1	700.3	0	2.2593
CL3652.Contig1	KY938828	UDP-xylose and UDP-N-acetylglucosamine transporter-like	*Bombyx mori*	XP_004925171.1	609.8	2.00E-172	2.2445
Unigene18177	KY938838	3-hydroxy-3-methylglutaryl coenzyme A reductase	*Helicoverpa armigera*	ADM13643.1	397.1	1.00E-108	2.1412
Unigene8998	KY924791	Arylphorin subunit beta-like	*Bombyx mori*	XP_004931864.1	134.8	1.00E-29	2.1163
CL3082.Contig1	KY963167	Membrane associated progesterone receptor	*Culex quinquefasciatus*	XP_001871002.1	229.2	3.00E-57	1.9096
Unigene6766	KY938820	Minus strand dynein intermediate chain	*Bombyx mori*	XP_004933487.1	407.5	3.00E-112	1.5108
CL483.Contig1	KY938829	Transmembrane protein adipocyte-associated 1 homolog	*Bombyx mori*	XP_004922477.1	646.7	0	1.1719
Unigene5246	KY827831	Vitellogenin	*Anthonomus grandis*	AAA27740.1	211.8	2.00E-53	1.1396
Unigene29549	KY938825	Methoprene tolerant protein 1	*Helicoverpa armigera*	AHX26585.1	194.9	1.00E-48	0.9643
Unigene7634	KY924782	Vitellogenin	*Anthonomus grandis*	AAA27740.1	121.7	1.00E-26	0.8357
Unigene6579	KY938836	Minus strand doublesex protein female specific variant	*Ostrinia scapulalis*	BAJ25851.1	278.1	1.00E-73	0.7697
Unigene37772	KY924781	Vitellogenin-2-like	*Bombyx mori*	XP_004926800.1	154.5	2.00E-36	0.5792
Unigene37397	KY938840	Period	*Plodia interpunctella*	AAC72329.1	255	1.00E-66	0.532
CL1945.Contig1	KY938813	Ecdysone receptor	*Plodia interpunctella*	AAR84611.1	970.3	0	0.125
CL1537.Contig1	KY938822	Electron transfer flavoprotein-ubiquinone oxidoreductase	*Bombyx mori*	XP_004925425.1	1138.6	0	0.1108

### Identification of *Vg* genes from *Cadra cautella* transcriptome data and validation by RT-PCR

**Fig. 7:**
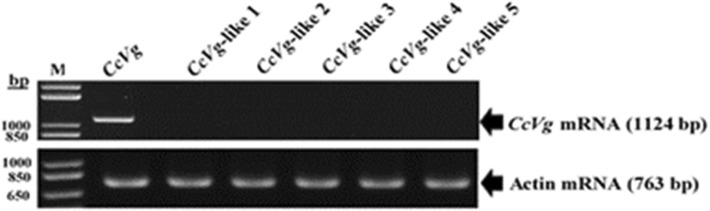
Confirmation of Cadra cautella Vg gene transcripts. Cadra cautella Vg gene transcripts identified by next- generation sequencing with reverse transcription polymerases chain reaction (RT-PCR). Agarose gel 1.2% was used to analyze the amplified PCR products. The CcVg and actin genes amplified products size are shown on the right. The amplified bands were visualized under ultra violet light and photographed using gel documentation BioDocAnalyze system (Biometra). M = molecular weight marker, bp = base pairs

The *C. cautella* transcriptome data provided six partial *Vg* gene transcripts. Among the six *Vg* transcripts, one of the transcripts was more highly expressed with a FPKM value 3234.95 than the other five *Vg* transcripts (FPKM values of 6.343, 3.34, 1.13, 0.83, and 0.057, respectively). These transcripts were designated as *CcVg*, *CcVg* like 1, *CcVg* like 2, *CcVg* like 3, *CcVg* like 4*,* and *CcVg* like 5. The information regarding the length, and compositions, of the 6 transcripts identified in the transcriptome assembly, are given in the Additional file 4: Table S3. It was very important to check how many of the *Vg* transcripts were functional in *C. cautella*. Therefore, the expression levels of all *Vg* transcripts were verified by RT-PCR using gene specific primers (Additional file 5: Table S4). The gene specific primers were designed based on the partial transcripts identified in the transcriptome assembly by using Primer3 software **(**http://bioinfo.ut.ee/primer3-0.4.0/). The amplified cDNA was sequenced and aligned by using (BioEdit Sequence Alignment Editor) with the 6 *Vg* transcripts, result showed that the amplified sequence was exactly similar with the partial sequence of *CcVg* transcript. It reflects that *CcVg* had a higher expression level (over 3000 times) than that of the other five *Vgs* transcripts, and it might be the primarily functional *Vg* gene in *C. cautella* (Fig. 7).

## Discussion

The order Lepidoptera is one of the most important groups of insect pests, which cause severe losses to agricultural products worldwide. The majority of lepidopterans (approximately 90%) are moths, with their caterpillars in particular being notorious pests of agricultural produce. Approximately 70% of moths are linked to stored product infestations. The almond moth, *C. cautella* (Walker), is an economically important pest of dates [6, 12, 45]. Recent studies have focused on its biology and ecology, and have proposed several management strategies to control these pests, including use of botanical extracts [46], heat treatments [47], freezing effects [48], essential oil extract [49, 50], and modified atmosphere [12, 51]. However, due to a lack of genetic information nothing is known about the reproductive mechanism of this economically important pest. Thus, the objective of the present study was to isolate the reproduction control genes from *C. cautella* by deploying the NGS approach.

Illumina NGS sequencing of *C. cautella* resulted in 62,687 unigenes discovered, with 44.4% of these (27836) having remarkable homology to operating genes encoding precise proteins with BLASTX analysis in GenBank. The analysis of unigenes homology indicated that 45.59% of genes showed the highest resemblance with *Bombyx mori* followed by 31% similarity with *D. plexippus*. These results indicate that *C. cautella* has a closer relationship to *Bombyx mori* and *D. plexippus* then to other lepidopteran members [52]. *Bombyx mori* is an extremely significant model organism for insect biology, in particular, and other life sciences, in general. The species distribution of *C. cautella* unigenes was almost in accordance with the transcriptome analysis results of other lepidopteran species such as *Galleria mellonella* and *Heliothis virescens* [53, 54].

In the present study, 8828 unigenes were annotated using the COG database. In COG analyses, the most frequently identified class was related to the general function prediction, followed by replication, recombination and repair, translation, ribosomal structure and biogenesis, function unknown, transcription, and post translational modification (Fig. 3). The general function prediction class (3636 unigenes, 41.18%) was the largest COG class, which was similar to the results of Shen, Dou [42] and Yan, Liu [55].

We surveyed our transcriptome data and identified several important enzymes and genes involved in reproduction. The *Vg*, *VgR*, lipophorin, lipophorin receptor, apolipophorins, doublesex, transmembrane protein, juvenile hormone esterase, ecdysone oxidase, rab5, and many others were identified (Table 3). In the present study, 57 genes encoding proteins vital for reproduction have been submitted to the NCBI genomic database and their accession numbers obtained (Table 3).

The *Vg* gene play a major role in insect reproduction and proliferation. The specificity of *Vg* with sex, tissues, and stage has been reported in many insect species [30, 56]. *Vg* gene expression in female fat body tissues and the evidence of Vg protein in adult female hemolymph and ovariole extracts have been reported in the American cockroach *Periplaneta americana* [21], madeira cockroach, *Leucophae maderae* [28], and oriental leafworm moth, *Spodoptera litura* [57]. It has been reported that different insect species have different numbers of *Vg* genes [58]. In some insects, there is one *Vg* gene, whereas others have two or multiple *Vg* genes. Multiple *Vg* genes have been described from numerous insect species including *Aedes aegypti* [59], brown winged green bug, *Plautia stali* [60], *Periplaneta americana* [21, 27], and *Leucophae maderae* [28, 29]. However, in the lepidopteran species till date only one *Vg* transcript has been reported which might yield different numbers of yolk polypeptides as identified in pyralid moths including *C. cautella* comprising two true vitellogenin subunits (+/−160KDa and 47KDa) [61]. Whereas, we reported only one functional vitellogenin transcript in the present data, because there are post transcriptional modifications and the *Vg* transcript cleaves into two subunits of different size and in this regards the two polypeptides of different size can bee observed. Previously we have cloned and characterized several vitellogenin and its receptors genes in different insect species and reported very clearly about the cleavage process of *Vg* transcript in insects. For detail plz. See [28, 56, 58].

To date, the complete Vg mRNA has been sequenced from 23 lepidopteran species; however, among these there is only one species, the rice moth *Corcyra cephalonica* that is associated with stored grain infestations. Thus, the addition of *C. cautella* Vg/VgR and other transcripts in the GenBank will strengthen the amount of available genomic data regarding reproductive physiology. Additionally, to date there is no report on the sequencing of the VgR from any moth species, which is also associated with stored grain infestations. The Vg protein is carried by the hemolymph to the ovaries where it is taken up by its counterpart, the VgR, and deposited in the developing oocyte. The VgR is an important carrier for the uptake of Vg into the developing oocytes of all oviparous species [58]. The VgRs of insects have large membrane bound proteins approximately 180–214 kDa in size [62]. The molecular characterization of insect VgRs has revealed that these receptors, regardless of their origin, are extremely conserved not only in their structure but also in terms of their regulation [21, 63]. VgR plays a crucial role in insect reproduction but little is known about this receptor in insects compared to its ligand, the Vg.

The higher expression of some transcripts in the *C. cautella* adult female abdominal tissue has revealed the importance of these genes in the biological process, physiology, and reproduction of *C. cautella*. Vg and apolipophorin are very important for the nourishment of the developing embryo inside the egg. Yolk polypeptide 2, follicular epithelium yolk protein, and ribosomal proteins were among the most abundant transcripts, which play crucial roles in the reproduction of insects [64, 65]. Similarly, lipid carrier protein (apolipophorins) and sulfur containing amino acids carrying proteins (hexamerins), might play a role to enhance the vitellogenesis, whereas, the egg shell protein (chorion), juvenile hormone, and ecdysteroids play crucial roles in insect metamorphosis and reproduction [66–70]. In the desert locust, the silencing of ecdysone receptor affected the choriogenesis and ovarion development. The effect was not only limited up to the disruption of oogenesis it also has affected the JH biosynthesis in corpora allata [71]. Insect development and reproduction are primarily associated with the fluctuating levels of these genes [72, 73]. Methoprene tolerant protein, works as a nuclear receptor for the JH functioning and plays a key role in the larval metamorphosis as well as vitellogenesis in adult females. In *H. armigera*, the knockdown of methoprene tolerant gene has adversely affected the larval development and adult female oogenesis [74].

The present study is the foremost distinctive study that has provided a wealth of genes related to molecular mechanism of reproduction in *C. cautella*, which is the key pest of stored grains and dates.

## Conclusions

The warehouse moth, *C. cautella*, is a serious pest of dates, both in the field and under storage conditions. The present study provides comprehensive data on reproduction control genes including *Vg* that has vital importance and the genes expressed in the abdominal tissues related to different physiological functions such as juvenile hormone and ecdysone receptor. Results from the present study have greatly strengthened the genetic understanding of different life processes of this pest. The availability of a huge number of transcripts will provide a foundation for future studies. Although NGS data provided 6 CcVg partial transcripts, RT-PCR analysis together with high expression level identified in terms of FPKM values showed that there might be one functional *Vg* gene (*CcVg*) in *C. cautella*. Next efforts will be made to get full sequence of these genes, their characterization, expression analysis, and knockdown deploying RNAi technology. The sequencing/characterization and silencing of reproduction control genes will elucidate the developmental strategies of *C. cautella* at the molecular level and, it could lead toward the development of an environmentally benign strategy for the management of this key pest.

## Methods

### Insect rearing

The *C. cautella* culture was maintained at the Economic Entomology Research Unit, Department of Plant Protection, College of Food and Agriculture Sciences, King Saud University, Riyadh, Saudi Arabia. The colony was maintained in an environmental chamber (Steridium, Australia) at 25 ± 2 °C and 65 ± 5% relative humidity under a 15:9 (light/dark cycle) on a slightly modified artificial diet media developed by [75]. Wandering instar larvae were separated to pupate and female pupae were placed separately in the growth chamber under the same conditions as those for the tissue collection.

### Tissue preparation for transcriptome analysis

One-day old virgin adult *C. cautella* females were selected for tissue preparation because from the previous studies, it is obvious that the expression of vitellogenin receptor and its ligand remains maximum in one-two days old females confirmed through semi quantitative and qRT-PCR results of several studies. In silk moth, the maximum expression of Vg was reported at the age of 24 h old female moth. Several other studies have also reported the same findings in lepidopteran species [28, 31, 57, 66]. The female moths were hold gently, their wings were removed and the last 5–7 abdominal segments were cut out with micro scissors and placed directly into phosphate buffered saline (PBS; pH 8.0) solution for washing [28]. Tissues were washed for 3–4 min in the PBS solution, and then transferred into a 1 mL Eppendorf tube containing liquid nitrogen, and preserved at − 80 °C until subsequent analysis.

### RNA isolation and construction of cDNA library for transcriptome analysis

The abdominal tissues of one-day-old virgin female moths ~ 800 mg in size were used for RNA extraction with Tri-RNA reagent (Favorgen Biotech CORP, Taiwan). The total RNA concentration, RNA integrity number, 28S/18S, and size of the RNA sample were determined using an Agilent 2100 bioanalyzer and Agilent RNA 6000 nano kit, and the purity of the sample was assessed using a nanodrop instrument. The concentration and total volume of the RNA samples were 378 ng/μL and 70 μL, respectively, and the RNA integrity number was 5.2. The integrity of RNA was confirmed by 1% agarose gel electrophoresis.

After the confirmation of RNA integrity, total RNA was used for cDNA synthesis. The total RNA sample was digested by DNaseІ (New England Biolab), purified by oligo-dT beads (Dynabeads mRNA purification kit, Invitrogen), and then poly (A)-containing mRNA were fragmented into 130 base pairs (bp) with the first-strand buffer. First-strand cDNA was generated by random hexamer primers (N6), first-strand master mix, and super script II reverse transcription (Invitrogen) (reaction conditions: 25 °C for 10 min, 42 °C for 50 min, and 70 °C for 15 min).

For the second-strand cDNA synthesis, a second-strand master mix was added to the first-strand cDNA and the prepared mixture was incubated for 1 h at 16 °C. AMPure XP magnetic beads were used to purify the double strand cDNA. The purified cDNA was subjected to the End-Repair mix to recover any damaged or incompatible ends, incubated for 30 min at 20 °C, and then purified. The products were ligated with one another using a sequencing adapter and, after agarose gel electrophoresis, a suitable size range of fragments were selected for polymerase chain reaction (PCR) amplification with a PCR primer cocktail and PCR master mix at 20 °C for 20 min. Finally, PCR products were purified using AMPure XP beads, the library was quantitated, and the qualified libraries were sequenced using the Illumina HiSeqTM 2000 system.

### Illumina sequencing and de novo assembly

The library was quantitated and the qualified libraries were amplified on cBot to generate clusters on the flow cell (TruSeq PE Cluster Kit V3–cBot–HS Illumina), and the amplified flow cell was sequenced pair end on the HiSeq 2000 system (TruSeq SBS KIT-HS V3, Illumina). The sequences with a read length of 50 bp were sequenced with a paired end strategy (Additional file 2: Figure S1). Raw reads produced from the sequencing machine contain dirty reads composed of adopters, which are unknown or low-quality bases that have a negative effect on the bioinformatics analysis. Therefore, the raw reads produced from the sequencing data were cleaned by removing the reads with adopters and reads with unknown nucleotides larger than 5% with the help of filter-fq software (version internal filter_fq software of BGI). Transcriptome de novo assembly was carried out with the short reads assembling program Trinity (version = release-20,130,225) [76]. Further, the TGICL (version = v2.1) and Phrap (version = Release 23.0) software were used for the downstream processing of large volumes RNA-sequence reads into unigenes.

### Unigenes annotation and functional organization

In the final step, unigenes were aligned to the nucleotide database (NT) with the blastN and protein databases: non-redundant protein (NR), Swiss-Prot, Kyoto Encyclopedia of Genes and Genomes (KEGG), and clusters of orthologous groups of protein (COG) via BLASTX with an e-value < 0.00001. The sequence alignment outcomes with greatest sequence resemblance were selected and annotated to unigenes. The unigenes that were unsuccessful in lining up the above mentioned databases were separated out with ESTScan software to decide sequence direction and detect coding region. Blast2GO software (version = v2.5.0) was used in NR annotation to obtain gene ontology (GO) annotation (i.e., biological process, molecular function, and cellular component) [77].

WEGO software was applied to deduce the functional classification of all annotated unigenes [78]. All unigenes were aligned with the COG database to classify and investigate their possible functions. Similarly, the KEGG pathway database was surveyed with the BLASTX program to predict the possible pathways where each of the unigenes were involved.

### Validation *Vg* gene transcripts via reverse transcription (RT) PCR

Six transcripts of the *Vg* gene with dissimilar fragments per kilobase of transcript per million mapped reads (FPKM) values were recognized from the *C. cautella* female abdominal tissue transcriptome. The 6 *Vg* transcripts were evaluated through RT-PCR with gene specific primers synthesized from the 6 transcripts they had identified in the transcriptome assembly. Actin gene primers, *Cc-*Act-F1 and *Cc-*Act-R1, were used as internal controls (Additional file 5: Table S4). For validation, a cDNA library was exposed to PCR with the Gene Amp PCR system 9700 thermo cycler (Applied Biosystems, Foster City, CA, USA), and the following PCR conditions were used: initial denaturation at 94 °C for 1 min, followed by 32 cycles of denaturation at 94 °C for 30 s, and annealing at 68 °C for 3 min. The PCR-amplified products were run on 1.2% agarose gel, stained with ethidium bromide for 30 min, and visually observed under ultra violet light with the gel documentation system BioDocAnalyze (Biometra). The successful amplified samples were sent to BGI for sequencing.

## Supplementary information

**Additional file 1: Table S1.** Summary statistics of *Cadra cautella* adult female abdominal tissue transcriptome.

**Additional file 2: Figure S1.** Diagrammatic view of assembly process from raw reads to contigs and unigene clustering.

**Additional file 3: Table S2.** All-unigenes blast CDs represent the protein-coding sequences mapped to the protein database. All-unigene EST scan CDs represent the protein-coding sequences that were predicted by ESTScan.

**Additional file 4: Table S3.** Summary of length and composition of the 6 *Vg* transcripts identified in the transcriptome assembly of *Cadra cautella* adult female abdominal tissues.

**Additional file 5: Table S4.** List of primers used for confirmation of identified CcVg genes with RT-PCR.

## Data Availability

The transcriptome data of *C. cautella* female abdominal tissue has been submitted to the National Center for Biotechnology Information (NCBI) (accession no: SRP156514) and is freely accessible.

## Abbreviations

BGI: Beijing Genomics Institute
BLAST: Basic Local Alignment Search Tool
*CcVg*: *Cadra cautella* vitellogenin
CDS: Coding DNA sequence
COG: Clusters of Orthologous Groups of protein
EERU: Economic Entomology Research Unit
FPKM: Fragments per kilobase of transcript per million mapped reads
GO: Gene Ontology
KEGG: Swiss-Prot, Kyoto Encyclopedia of Genes and Genomes
NCBI: National Center for Biotechnology Information
NGS: Next-generation sequencing
NR: Non-redundant protein
NT: Non-redundant nucleotide
RT-PCR: Reverse transcription polymerase chain reaction
