# Supplementary material for: Transcriptome analysis of the almond moth, Cadra cautella, female abdominal tissues and identification of reproduction control genes
Source: BMC Genomics. 2019 Nov 21;20:883. doi: 10.1186/s12864-019-6130-2 (PMC6869320; doi:10.1186/s12864-019-6130-2)
Supplement: Supplementary file 1 — Additional file 1: Table S1. Summary statistics of Cadra cautella adult female abdominal tissue transcriptome. [file 12864_2019_6130_MOESM1_ESM.docx]

**Additional file 1: Table S1**

| **Table S1.** Summary statistics of *Cadra cautella* adult female abdominal tissue transcriptome | | | | | |
| --- | --- | --- | --- | --- | --- |
| **Output Statistics of Sequencing** | | | | | |
| **Total Raw Reads** | **Total Clean Reads** | **Total Clean Nucleotides (nt)** | **Q20 percentage** | **N percentage** | **GC percentage** |
| 113,775,104 | 108,942,268 | 9,804,804,120 | 98.40% | 0.01% | 43.76% |

| **Statistics of Assembly Quality** | | | | | | | |
| --- | --- | --- | --- | --- | --- | --- | --- |
|  | **Total Number** | **Total Length(nt)** | **Mean Length(nt)** | **N50** | **Total Consensus Sequences** | **Distinct Clusters** | **Distinct Singletons** |
| Contig | 106,031 | 43,406,915 | 409 | 1104 | - | - | - |
| Unigene | 62,687 | 55,234,116 | 881 | 1788 | 62,687 | 23,182 | 39,505 |
