# Supplementary figures and images for: Transcriptome analysis of the almond moth, Cadra cautella, female abdominal tissues and identification of reproduction control genes
Source: BMC Genomics. 2019 Nov 21;20:883. doi: 10.1186/s12864-019-6130-2 (PMC6869320; doi:10.1186/s12864-019-6130-2)

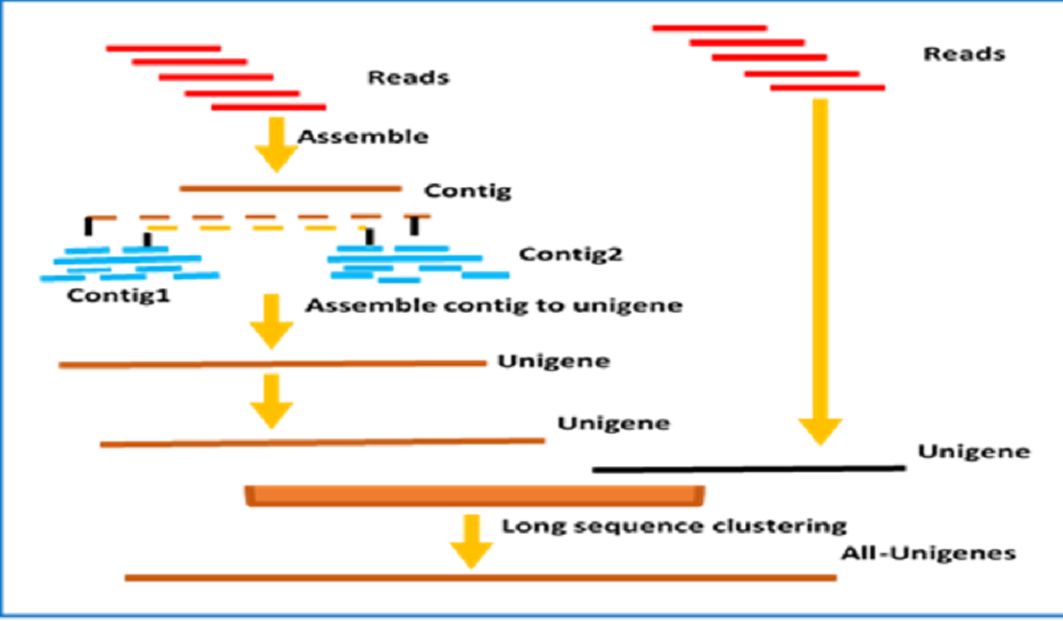

Supplement: Supplementary file 2 — Additional file 2: Figure S1. Diagrammatic view of assembly process from raw reads to contigs and unigene clustering. [file 12864_2019_6130_MOESM2_ESM.tif]
