# Supplementary material for: Transcriptome analysis of the almond moth, Cadra cautella, female abdominal tissues and identification of reproduction control genes
Source: BMC Genomics. 2019 Nov 21;20:883. doi: 10.1186/s12864-019-6130-2 (PMC6869320; doi:10.1186/s12864-019-6130-2)
Supplement: Supplementary file 3 — Additional file 3: Table S2. All-unigenes blast CDs represent the protein-coding sequences mapped to the protein database. All-unigene EST scan CDs represent the protein-coding sequences that were predicted by ESTScan. [file 12864_2019_6130_MOESM3_ESM.docx]

**Additional file: 3 Table S 2**

**Table S 2.** All-unigenes blast CDs represent the protein-coding sequences mapped to the protein database. All-unigene EST scan CDs represent the protein-coding sequences that were predicted by ESTScan.

| Sequence file | Sequence number |
| --- | --- |
| All-unigenes blast CDs | 25 715 |
| All unigene ESTScan CDs | 2719 |
| Total | 28 434 |
