# Supplementary material for: Transcriptome analysis of the almond moth, Cadra cautella, female abdominal tissues and identification of reproduction control genes
Source: BMC Genomics. 2019 Nov 21;20:883. doi: 10.1186/s12864-019-6130-2 (PMC6869320; doi:10.1186/s12864-019-6130-2)
Supplement: Supplementary file 4 — Additional file 4: Table S3. Summary of length and composition of the 6 Vg transcripts identified in the transcriptome assembly of Cadra cautella adult female abdominal tissues. [file 12864_2019_6130_MOESM4_ESM.docx]

Additional file 4: Table S 3

**Table S 3**. Summary of length and composition of the 6 *Vg* transcripts identified in the transcriptome assembly of *Cadra cautella* adult female abdominal tissues

| ***Vg* transcripts** | **A** | **T** | **G** | **C** | **G-C %** | **Length (nt)** |
| --- | --- | --- | --- | --- | --- | --- |
| *Cc*Vg | 1582 | 1245 | 1106 | 1389 | 46.9 | 5322 |
| *CcVg* like 1 | 1088 | 716 | 672 | 761 | 44.3 | 3237 |
| *CcVg* like 2 | 238 | 199 | 261 | 271 | 54.9 | 969 |
| *CcVg* like 3 | 164 | 139 | 117 | 105 | 42.3 | 525 |
| *CcVg* like 4 | 66 | 56 | 86 | 92 | 59.3 | 300 |
| *CcVg* like 5 | 91 | 63 | 51 | 63 | 42.7 | 268 |
