# Supplementary material for: Transcriptome analysis of the almond moth, Cadra cautella, female abdominal tissues and identification of reproduction control genes
Source: BMC Genomics. 2019 Nov 21;20:883. doi: 10.1186/s12864-019-6130-2 (PMC6869320; doi:10.1186/s12864-019-6130-2)
Supplement: Supplementary file 5 — Additional file 5: Table S4. List of primers used for confirmation of identified CcVg genes with RT-PCR. [file 12864_2019_6130_MOESM5_ESM.docx]

Additional file 5: Table S 4

| Primers | Sequences |
| --- | --- |
| *Cc*Vg-F | 5ʹ GTGATGAAGTGAGGCAGCAACAGTA 3ʹ |
| *Cc*Vg-R | 5ʹ GAGCCTGATTGAGGACCTTTGTGTA 3ʹ |
| *Cc*Vg1-F | 5ʹ TCATCTACCTCTTTCGGCTGATGTC 3ʹ |
| *Cc*Vg1-R | 5ʹ AGAGTCAGTCAGATCCAAACCGTTG 3ʹ |
| *Cc*Vg2-F | 5ʹ CCAGACCAGCATGATAGATATGACCA 3ʹ |
| *Cc*Vg2-R | 5ʹ GTCGTCAAACAGGTTGCAGATTAGG 3ʹ |
| *Cc*Vg3-F | 5ʹ CCATGGCTAAGTCTGTTCGTACACC 3ʹ |
| *Cc*Vg3-R | 5ʹ TCCATCAACCTCATCAAAGAGGAAA 3ʹ |
| *Cc*Vg4-F | 5ʹ TTCGTAGTCCAGCAAGGCTTAGACC 3ʹ |
| *Cc*Vg4-R | 5ʹ GGTCTAAGCCTTGCTGGACTACGAA 3ʹ |
| *Cc*Vg5-F | 5ʹ CATGAACATGAACCAACTGCCAATA3ʹ |
| *Cc*Vg5-R | 5ʹ CTTCGCTGTTGGTGTAGTTCTTGCT |
| Act*Cc-*F | 5ʹ GTCGGTATGGGTCAGAAAGACTCCT 3ʹ |
| Act*Cc-*R | 5ʹ TACATGGTGGTACCTCCGGACAATA 3ʹ |

**Table S 4.** List of primers used for confirmation of identified *CcVg* genes with RT-PCR
